# Evidence of Genomic Exchanges between Homeologous Chromosomes in a Cross of Peanut with Newly Synthetized Allotetraploid Hybrids

**DOI:** 10.3389/fpls.2016.01635

**Published:** 2016-11-01

**Authors:** Joel R. Nguepjop, Hodo-Abalo Tossim, Joseph M. Bell, Jean-François Rami, Shivali Sharma, Brigitte Courtois, Nalini Mallikarjuna, Djibril Sane, Daniel Fonceka

**Affiliations:** ^1^Centre d’Etudes Régional pour I’Amélioration de I’Adaptation à la SécheresseThies, Senegal; ^2^Département de Biologie et Physiologie Végétales, Université de Yaoundé IYaoundé, Cameroon; ^3^UMR AGAP, Centre de Coopération Internationale en Recherche Agronomique pour le DéveloppementMontpellier, France; ^4^International Crops Research Institute for the Semi-Arid TropicsPatancheru, India; ^5^Département de Biologie Végétale, Université Cheikh Anta DiopDakar, Senegal

**Keywords:** genetic map, disomic, polysomic, breeding, inheritance, peanut, allotetraploid

## Abstract

Cultivated peanut and synthetics are allotetraploids (2*n* = 4*x* = 40) with two homeologous sets of chromosomes. Meiosis in allotetraploid peanut is generally thought to show diploid-like behavior. However, a recent study pointed out the occurrence of recombination between homeologous chromosomes, especially when synthetic allotetraploids are used, challenging the view of disomic inheritance in peanut. In this study, we investigated the meiotic behavior of allotetraploid peanut using 380 SSR markers and 90 F_2_ progeny derived from the cross between *Arachis hypogaea* cv Fleur 11 (AABB) and ISATGR278-18 (AAKK), a synthetic allotetraploid that harbors a K-genome that was reported to pair with the cultivated B-genome during meiosis. Segregation analysis of SSR markers showed 42 codominant SSRs with unexpected null bands among some progeny. Chi-square tests for these loci deviate from the expected 1:2:1 Mendelian ratio under disomic inheritance. A linkage map of 357 codominant loci aligned on 20 linkage groups (LGs) with a total length of 1728 cM, averaging 5.1 cM between markers, was developed. Among the 10 homeologous sets of LGs, one set consisted of markers that all segregated in a polysomic-like pattern, six in a likely disomic pattern and the three remaining in a mixed pattern with disomic and polysomic loci clustered on the same LG. Moreover, we reported a substitution of homeologous chromosomes in some progeny. Our results suggest that the homeologous recombination events occurred between the A and K genomes in the newly synthesized allotetraploid and have been highlighted in the progeny. Homeologous exchanges are rarely observed in tetraploid peanut and have not yet been reported for AAKK and AABB genomes. The implications of these results on peanut breeding are discussed.

## Introduction

Allopolyploids and autopolyploids are two different types of polyploids, each resulting from a different genetic origin and showing a distinct meiotic behavior (Xu et al., 2015). In autopolyploids, all chromosome sets are identical or very closely related, while allopolyploids have divergent chromosome sets (Leitch and Leitch, 2008; Soltis et al., 2014). Sets of homologous chromosomes are considered “homeologous” to other sets from the other genomes (Sybenga, 1975, Sybenga, 1996). Thus, in classic autopolyploids, each chromosome may pair randomly with any of its homologs in equal frequencies during meiosis (Muller, 1914), leading to a polysomic inheritance. Autopolyploids can undergo double reduction where the segments of two sister chromatids end up in the same gamete (Haynes and Douches, 1993; Mather, 1936). In contrast to the situation in autopolyploids, the cytological diploidization of allopolyploids requires a nonrandom assortment of chromosomes into pairs, of which crossovers are exclusively formed between homologous chromosomes with disomic inheritance at each locus (Cifuentes et al., 2010; Gaeta and Chris Pires, 2010). However, in addition to these extremes, an intermediate pattern of genetic inheritance has also been described (Stebbins, 1947; Sybenga, 1996; Stift et al., 2008; Jeridi et al., 2012).

Cultivated peanut, *Arachis hypogaea* L., is one of the major oilseeds and cash crops worldwide for which genetic improvement can tremendously benefit from its wild relatives (Rami et al., 2014). This species is autogamous and allotetraploid (2*n* = 4*x* = 40), harboring homeologous A and B genomes (Husted, 1936; Smartt et al., 1978). It is assumed that it originated from a single hybridization event between two wild diploid taxa (Simpson et al., 2001), most likely *Arachis duranensis* (A genome) and *Arachis ipaensis* (B genome), followed by a spontaneous chromosome duplication (Seijo et al., 2004, 2007; Bertioli et al., 2016). The single origin of the crop, superimposed with domestication, resulted in a severe genetic bottleneck. Therefore, closely related diploid wild species, which have maintained a high genetic diversity, were considered suitable to broaden the genetic basis of the cultivated gene pool (Simpson, 2001; Favero et al., 2006; Foncéka et al., 2009). The wild relatives of cultivated peanut are mostly diploid (2*n* = 2*x* = 20) and contain species with A, B, D, K, and F genomes (Smartt et al., 1978; Stalker, 1991; Robledo and Seijo, 2010).

Given the ploidy difference between the wild and cultivated peanut, the production of colchicine-induced allotetraploids was used as a pathway to introduce wild alleles into the cultivated gene pool (Simpson, 1991). Several synthetic allotetraploids that have been produced by crossing different diploid species have proven to be cross-fertile with *A. hypogaea* (Mallikarjuna et al., 2011). Moreover, the development of peanut genomics tools has made possible the marker-assisted introgression of wild genes into a cultivated background (Fonceka et al., 2012).

However, this breeding approach has raised new fundamental questions on the meiotic behavior of the synthetic allotetraploid used in breeding programs and the possible genetic changes related to their genomic composition. Meiotic instabilities are common in interspecific and resynthesized lines (Gaeta et al., 2007; Lyrene, 2016). In some allopolyploid plants, recombination between subgenomes during meiosis was suspected to occur in newly formed polyploids (Ramsey and Schemske, 2002; Soltis et al., 2010) but was rarely observed among stabilized allopolyploids (Salmon et al., 2010; Ainouche and Wendel, 2014).

Based on the classic genetic behavior of the allotetraploid genome and cytogenetic observations, several genetic mapping studies in peanut have been conducted considering a diploid-like behavior at meiosis (Burow et al., 2001; Hong et al., 2008, 2010; Varshney et al., 2008; Foncéka et al., 2009; Qin et al., 2011; Shirasawa et al., 2013; Zhou et al., 2014). However, recently, thanks to a thorough analysis of genotyping data, Leal-Bertioli et al. (2015) reported unexpected missing and rare single nucleotide polymorphism (SNP) genotypes in recombinant inbred lines derived from a cross between a cultivated peanut and a synthetic allotetraploid. The authors showed that these missing data could be explained by the occurrence of partial tetrasomic recombination. Recombination among homeologous chromosomes is poorly understood in tetraploid peanut and the exact types of meiotic behavior remain unclear although the determination of these factors is important for our knowledge and for the development of appropriate breeding strategies. In addition, the classical impact of non-disomic inheritance on the genomic structure, such as segregation distortion and double reduction have not yet been reported.

In this study, we investigated the meiotic behavior of tetraploid peanut based on the segregation patterns of 380 microsatellites markers in F_2_ progeny derived from the cross between the cultivated peanut *Arachis hypogaea* and a synthetic allotetraploid *(Arachis duranensis* × *Arachis batizocoi)^4x^*. To obtain more insight into the mode of inheritance at the genome scale, we analyzed recombination events between homologous and homeologous chromosomes in relation to their position on a genetic linkage map. We reported the occurrence of a mixture of disomic and polysomic modes of inheritance of SSR loci, confirming the recent partial tetrasomic assumption made by Leal-Bertioli et al. (2015). We showed that this mixed inheritance was consistently associated with segregation distortion and homeologous chromosome substitution in some progeny. Our results suggest that the homeologous recombination events occurred between the A and K genomes in the newly synthesized allotetraploid and have been highlighted in the progeny.

## Materials and Methods

### Plant Material and Population Development

The study was conducted using an F_2_ population derived from the cross between the cultivated variety Fleur 11 and the synthetic allotetraploid ISATGR 278-18 *(Arachis duranensis* × *Arachis batizocoi)^4x^*. Fleur 11 is a Spanish type with erect growth habit widely cultivated in West Africa. ISATGR 278-18 was developed and kindly provided by ICRISAT-India (Mallikarjuna et al., 2011). The synthetic allotetraploid combines the AA genome of *A. duranensis* (ICG 8138; 2*n* = 2*x* = 20), a close wild relative and one of the most probable ancestors of *A. hypogaea*, and the KK genome of *A. batizocoï* (ICG 13160; 2*n* = 2*x* = 20), a wild relative taxa that was reported to pair with the B genome of the cultivated species during meiosis (Burow et al., 2001; Mallikarjuna et al., 2011; Leal-Bertioli et al., 2014).

ISATGR 278-18 was reported to have a normal chromosome configuration with 20 bivalents (Mallikarjuna et al., 2011). However, several cycles of self-pollination were performed at CERAAS prior to hybridization with Fleur 11. Five plants of the synthetic allotetraploid were used as male to cross with five plants of Fleur 11 used as female. The F_1_ plants were differentiated from plants derived from self-pollination of Fleur 11 using morphological traits (dark green leaves and procumbent growth habit). The F_2_ progeny were produced from the self-pollination of 15 F_1_ plants. All crosses were performed in plastic pots under greenhouse conditions at the Centre d’Etudes Régional pour l’Amélioration de l’Adaptation à la Sécheresse (CERAAS) in Senegal during 2011 and 2013.

### SSR Marker Analysis

Genomic DNA of both parents and F_2_ progeny was extracted from young leaves according to the MATAB protocol as described by Foncéka et al. (2009). Polymorphisms were assessed in the parents using 602 primer pairs, mainly selected from a previous study (Foncéka et al., 2009). The parents and F_2_ progeny were genotyped with polymorphic SSR markers. PCR was carried out in 96-wellplates in a total volume of 10 μl consisting of 5 μl of 5 ng/μl of the DNA template and 5 μl of a mixture of 0.1 μM of each SSR primer, 0.2 mM of each dNTP, 1X PCR buffer, 2.5 mM MgCl2, 0.1U/μl of Taq polymerase and 0.1 mM of IR700 or IR800-labeled M13 primer (MWG Germany) for fluorescence detection of SSR amplicons. A forward primer pair was labeled with a fluorescent (LI-COR Biosciences). Reactions were performed in an Eppendorf Mastercycler epgradient thermocycler. The PCR products were separated by electrophoresis run at a constant 95 W for 1-2 h in a DNA Sequencer (LI-COR 4300 DNA Analyzer, Lincoln, NE, USA).

Scoring of the SSR bands was performed visually on electrophoresis profiles using the application Jelly 2.017b (Rami, unpublished). For a codominant marker in an F_2_ population, we denoted “A” as the genotype of cultivated parent, “B” as the genotype of wild parent, and “H” as the genotype of their heterozygous hybrids. When one marker was dominant, the two non-separated genotypes “H” and “A” were denoted “D” and the two non-separated genotypes “H” and “B” were denoted “C”. For each SSR, the sub-genomic origin (A or K) of the wild alleles was assigned by comparing them to the alleles of the wild diploid progenitors. The sub-genomic origin of the cultivated allele was inferred by analyzing its co-inheritance with the wild alleles in the F_2_ progeny. Loci were suffixed by <A> or <B> for differentiating subgenomic origin. In all scenarios, missing data were scored as “x” and the unexpected null bands were scored as “N”.

### Meiotic Behavior Analysis

Based on the genotyping data for each SSR marker, we analyzed the genotype of the F_2_ progeny and deduced the allelic constitution of the gamete produced by the F_1_ hybrid. The genome of the cultivated species was noted “A_1_A_1_BB” and that of the wild species “A_2_A_2_KK”. Chromosomes “A_1_” and “A_2_” and “B” and “K” are homologous, while chromosomes “A_1_” and “B”, “A_1_” and “K”, “A_2_” and “B”, and “A_2_” and “K” are homeologous.

When recombination occurred only between homologous genomes, for SSRs that amplified only one locus on a given sub-genome, two segregating bands were expected and the segregation ratio in the F_2_ progeny was 1A_1_A_1_: 2A_1_A_2_: 1A_2_A_2_ or 1BB: 2BK: 1KK. For SSR that amplified the two homeologous genomes up to four segregating bands were expected. The segregation ratio in the F_2_ progeny is shown in **Table 1**. In this case, the cultivated and wild parents produced each one type of gamete (A_1_B and A_2_K, respectively) and the F_1_ hybrid (A_1_A_2_BK) would produce 4 types of gametes (A_1_B, A_1_K, A_2_B and A_2_K).

**Table 1:** Phenotypes and genotypes expected under tetrasomic and disomic inheritance if a SSR primer pair marks the homeologous genomes of the allotetraploid parents.

	Expected					
	Polysomic inheritance			Disomic inheritance		
	Phenotypes	Genotypes	Frequency	Phenotypes	Genotypes	Frequency
1	A_1_B	A_1_A_1_BB	1/36 (2.5)^a^	A_1_B	A_1_A_1_BB	1/16 (5.6)
2	A_1_BK	A_1_A_1_BK, A_1_BBK, A_1_BKK	6/36 (15)	A_1_BK	A_1_A_1_BK	2/16 (11.3)
3	A_1_A_2_B	A_1_A_2_BB, A_1_A_1_A_2_B, A_1_A_2_A_2_B	6/36 (15)	A_1_A_2_B	A_1_A_2_BB	2/16 (11.3)
4	A_1_A_2_BK	A_1_A_2_BK	6/36 (15)	A_1_A_2_BK	A_1_A_2_BK	4/16 (22.5)
5	A_1_A_2_K	A_1_A_2_KK, A_1_A_2_A_2_K, A_1_A_2_A_2_K	6/36 (15)	A_1_A_2_K	A_1_A_2_KK	2/16 (11.3)
6	A_2_BK	A_2_A_2_BK, A_2_BBK, A_2_BKK	6/36 (15)	A_2_BK	A_2_A_2_BK	2/16 (11.3)
7	A_1_K	A_1_A_1_KK	1/36 (2.5)	A_1_K	A_1_A_1_KK	1/16 (5.6)
8	A_2_B	A_2_A_2_BB	1/36 (2.5)	A_2_B	A_2_A_2_BB	1/16 (5.6)
9	A_2_K	A_2_A_2_KK	1/36 (2.5)	A_2_K	A_2_A_2_KK	1/16 (5.6)
10	A_1_A_2_	A_1_A_1_A_2_A_2_	1/36 (2.5)	/	/	/
11	BK	BBKK	1/36 (2.5)	/	/	/

*^a^The expected frequency of the 90 F_2_ progeny is indicated in brackets.*

When recombination occurred between homeologous genomes, for SSRs that amplified only one locus on a given sub-genome, a fourth ‘null’ genotype is expected to appear in addition to the three expected genotypes in an F_2_ population. For SSRs that amplified the two loci, one on each of the homeologous genomes, 11 phenotypic bands are expected in the gel (**Table 1**). The segregation ratio in the F_2_ progeny depends on the genomic constitution of the F_1_ hybrids. For F_1_ with normal genomic constitution (without homeologous recombination during parental meiosis), the expected segregation ratio in the F_2_ progeny is shown in **Table 1**. It is not straightforward to estimate the genotypic frequencies in F_2_ progeny derived from “aberrant” F_1_ (resulting from homeologous recombination events during parental meiosis). In contrast to the homologous pairings, two specific phenotypes, A_1_A_2_ and BK, are observed in homeologous pairings. In this study, we use the terminology “homeologous-recombinant genotypes” for the progeny that carried these two peculiar phenotypes.

### Allele Number in the Homeologous Recombinant Genotypes

In the electrophoresis profiles, the band intensities of the homeologous-recombinant progeny were analyzed to determine the number of copies of each allele. The band intensities were determined using ImageJ version 1.46 (Ferreira and Rasband, 2012), and the dosage ratio between bands was compared to the relationships expected between alleles in the hypothetical configurations. For example, for the homeologous-recombinant progeny with A_1_A_2_ band-phenotype on the gels, three genotypes are possible with ratios of allelic combinations of 3:1 (A_1_A_1_A_1_A_2_), 1:1 (A_1_A_1_A_2_A_2_) or 1:3 (A_1_A_2_A_2_A_2_). The genotype was then determined based on allelic dosage. The F_2_ progeny with four different alleles (i.e., A_1_A_2_BK) were used as a reference for determination, as these progeny involve single-copy alleles.

### Segregation Analysis

The segregation of codominant and dominant loci was compared to the segregation ratios expected under disomic inheritance (1:2:1 for codominant loci and 3:1 for dominant loci), using the chi-square test of the software “Calculation for the chi-square test” (Preacher, 2001). Data scored as “N” (null phenotype) as well as missing data were not considered in the analysis. Loci that deviated significantly (*P* < 0.05) from the theoretically expected ratios were considered distorted and were represented by an asterisk on the genetic map.

### Linkage Analysis and Map Construction

Linkage analysis was performed using the MapDisto software (Lorieux, 2012) using only the co-dominant loci. The linkage map was constructed in several steps. In the first step, markers that showed distorted segregation (*P* < 0.05) and those with unexpected null data were excluded. The non-distorted loci were grouped into LGs using a minimum LOD of 3, a maximum recombination frequency *r* of 0.3 and the Kosambi mapping function (Kosambi, 1943). The order of markers within each linkage group (LG) was determined using the “order” and “ripple” commands. In a second step, the distorted loci and loci with unexpected null data were progressively added into the established LGs if their presence did not significantly affect the marker order. The position of the loci with unexpected null data was adjusted iteratively and the poorly mapped loci were removed. The synteny analysis between the homeologous genome was performed on the basis of the mapped homeologous loci (Foncéka et al., 2009). The graphical linkage maps were drawn using SpiderMap software (Rami, unpublished) and the graphical genotype was drawn using the GGT software (van Berloo, 2008).

## Results

### Polymorphism of the SSR Markers and Segregation of Parental Alleles in the F_2_ Progeny

Among the 602 SSR markers screened, 447 (74%) detected a polymorphism between the parental genomes. A total of 431 (71.6%) SSR loci were polymorphic between the A homologous genomes (A_1_ and A_2_) and 465 (77.2%) between the B/K homologous genomes. From these polymorphic SSR markers, 380 that provided accurate amplification profiles were analyzed in the F_2_ progeny and generated 562 loci, out of which 378 (67%) segregated as codominant and 184 (33%) segregated as dominant. Among the 378 codominant loci, 194 (51%) were assigned to the A-genomes and 184 (49%) were assigned to the B/K-genomes. Among the 184 dominant loci, 70 (38%) were from the A-genomes, whereas 114 (62%) were from the B/K-genomes.

The segregation of the SSRs was assessed with regard to their informativity for distinguishing polysomic versus disomic inheritance. This allowed for the classification of the SSR into ten classes (**Table 2**). The SSR markers within class 1 appeared as the most informative ones because they marked the homeologous genomes in both parents, allowing the identification of the expected genotypes for each of the meiotic behaviors in the F_2_ progeny. They were chosen to illustrate the type of inheritance.

**Table 2:** Segregation of parental alleles and informativeness of polymorphic SSR markers to distinguish polysomic vs. disomic inheritance in the F_2_ progeny.

Marker class	Cultivated parent	Wild Parent	Relative frequency (%)	Segregation in the F_2_ progeny		Informative polysomic vs. disomic inheritance
				A-genome	B-genome	
1	A_1_A_1_BB	A_2_A_2_ KK	25.1	<ABH>	<ABH>	Completely
2	A_1_A_1_BB	A_2_A_2_KK	1.4	<ABH>	none^a^	Highly
3	A_1_A_1_BB	A_2_A_2_ KK	11.3	None^a^	<ABH>	Highly
4	A_1_A_1_- -	A_2_A_2_ KK	7.9	<ABH>	<AC>	Moderately
5	A_1_A_1_BB	- - KK	3.4	<ABH>	<ABH>	Moderately
6	A_1_A_1_- -	A_2_A_2_- -	18.1	<ABH>		Partially
7	- -BB	- - KK	10.5		<ABH>	Partially
8^b^	A_1_A_1_- -	- - - -	2.0	<BD>		Lowly
9^b^	- - - -	- - KK	5.1		<AC>	Lowly
10	Duplicated		15.3			Lowly

*Genotypes were scored as “A”, “B”, and “H” for codominant loci and “B” and “D” (or “A” and “C”) for dominant loci. ^a^Indicates homologous alleles of identical size on the gel (monomorphism). ^b^Dominant allele could be provided either by the cultivated or the wild parents.*

### Inheritance Patterns of SSR Markers in the F_2_ Progeny

**FIGURE 1:**
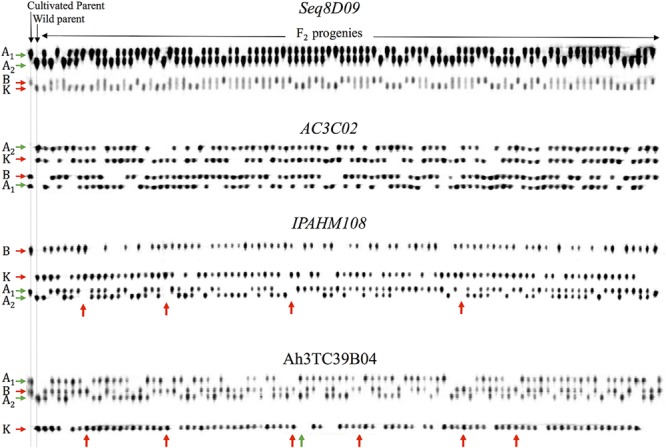
**Images of the segregation of four SSR markers.** The cultivated and wild parents are in lanes 1 and 2, respectively. The F_2_ progeny are distributed from lane 3 to the end. Each primer pair amplified two segregating bands in both parents. Each band (allele) was assigned to a subgenome figured with arrows on the left. For the Seq8D09 and AC3C02 markers, the homologous bands inherited as two independent codominant loci in the F_2_ progeny, i.e., A_1_A_2_ genotype at one locus and BK at the other, as expected under disomic inheritance. At IPAHM108 and Ah3TC39B04 markers, the red and green arrows show the homeologous-recombinant F_2_ progeny that own the BK and Aa by A_1_A_2_ respectively. The homologous bands of the A-genome (“A_1_” and “A_2_”) are lacking whereas the homologous bands of the B/K-genome are systematically observed in 4 and 6 progeny (red arrows). Reciprocally, the “B” and “K” bands are lacking in one F_2_ progeny, whereas, the “A_1_” and “A_2_” bands are observed (green arrow).

The segregation analysis of 380 SSR markers revealed that 338 (88.9%) were inherited in an F_2_ population, as expected under disomic behavior. Two compelling examples of disomic segregations are shown in **Figure 1** for the SSR markers Seq8D09 and AC3C02. These primer pairs marked the homeologous genomes in both parents and amplified up to four segregating bands. Each band was assigned to a subgenomic allele (“A_1_”, “B”, “A_2_” and “K”). The homologous bands of A-genomes (“A_1_” and “A_2_”) and B/K-genomes (“B” and “K”) are inherited as two independent codominant loci in the F_2_ progeny, Aa at one locus and Bb at the other (**Figure 1**).

Interestingly, 42 (11.1%) SSR markers exhibited several unexpected F_2_ band-phenotypes, which are impossible to explain under disomic behavior. Null bands for one of the homologous genomes were observed in some progeny, although the primer pair amplified the two homeologous genomes. Two examples of these unexpected F_2_ phenotypes are shown in **Figure 1** for the SSR markers IPAHM108 and Ah3TC39B04. These markers amplified up to 4 segregating bands. However, the homologous bands of the A-genomes (“A1” and “A2”) are lacking for some F_2_ progeny, whereas the homologous bands of the B/K-genomes (“B” and “K”) are observed, excluding the missing data assumption. Those F_2_ progeny are indicated with red arrows on **Figure 1**. Reciprocally, for the Ah3TC39B04 marker, the homologous bands of the B/K-genomes are missing, while the homologous bands of the A-genome are observed in one F_2_ progeny (marked with a green arrow on **Figure 1**). These A_1_A_2_- and BK-phenotypes are unexpected under disomic behavior, but are expected under polysomic behavior (**Table 1**). These phenotypes occurred if and only if the homologous alleles ended up in the same gamete during the parental and/or the F_1_ meiosis.

Remarkably, the value of the intensity of the “B” and “K” bands varied significantly among the BK-phenotypes for the SSR markers that segregated in a polysomic-like pattern, suggesting differences in the number of alleles. For example, in some BK-phenotypes, the more intense bands (“K”) represent two and three doses, while the less intense bands (“B”) represent a single dose (Supplementary Tables S1-S10). The K/B intensity ratio ranged from 1 to 3, indicating that the genotype of the BK F_2_ band-phenotypes was either BBKK (1:1) or BKKK (1:3). The BKKK genotype is unexpected in the F_2_ progeny derived from the A_1_A_2_BK F_1_ hybrid (**Table 1**). The presence of BKKK genotypes suggests the occurrence of homeologous recombination during the meiosis of the parents, particularly in the synthetic allotetraploid.

Moreover, for three polysomic SSR such as RN13D04, only one allele was observed in one F_2_ progeny whereas two alleles were present in both parents with up to four alleles segregating in the population (data not shown).

### Segregation Distortion Analysis

Among the 378 codominant loci scored in the F_2_ progeny, 336 showed disomic inheritance. Of these, 279 (83%) followed the 1:2:1 Mendelian segregation ratio expected in an F_2_ population and the remaining 57 (16%) loci deviated significantly from it (*P* < 0.05). A total of 17 and 40 loci were distorted for the A and B/K-genomes, respectively (**Table 3**). Of the 17 distorted loci for the A-genome, 14 were skewed toward the cultivated genotypes, whereas one and two loci were distorted in favor of the wild and heterozygous genotypes, respectively. Inversely, among the 40 loci distorted for the B/K-genome, 26 and 14 were skewed toward the wild and heterozygous genotypes, respectively. These results indicated differences between genomes and genotypes for SD.

**Table 3:** Segregation of the loci in the F_2_ population.

Types of segregation	Types of heredity			Total
	Disomic		Polysomic	
	A-Genome	B/K-Genome		
Codominant	167 (17)^a^	169 (40)	42 (42)	378
Dominant	70 (8)	114 (27)		

*^a^The number of distorted loci is indicated in parenthesis.*

All the 42 codominant SSR markers that showed an unexpected genotype under disomic inheritance were distorted when tested under the 1:2:1 Mendelian segregation ratio. We were not able to test for the segregation ratio expected under polysomic behavior (1:34:1) since the genomic constitution of the F_1_ hybrids was unknown and unexpected genotypes could result from “aberrant” F_1_ hybrids. Nonetheless, as indicated on the map below, these codominant distorted markers were clustered in particular regions on different LG, suggesting that such distortion likely originated from homeologous pairing.

### Map Construction and Linkage Analysis

**FIGURE 2:**
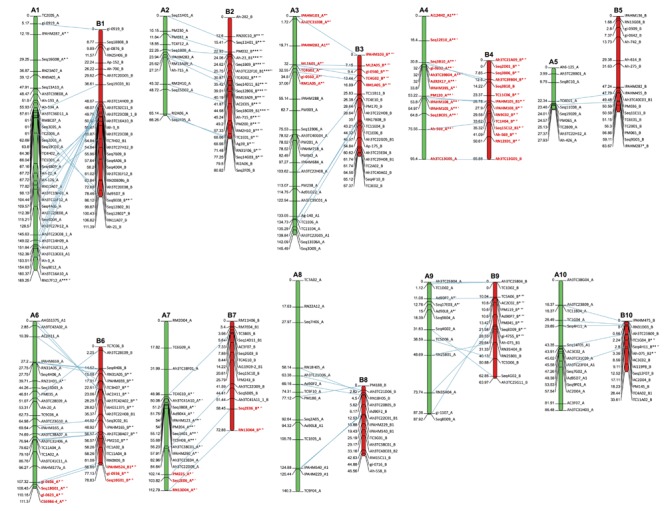
**Genetic linkage map based on 90 F_2_ progeny and distribution of the disomic and polysomic codominant loci in the linkage groups (LG).** The green and red segments indicate the LGs deriving from the A genome, named from A1 to A10 and the B genome named from B1 to B10, respectively. The map distances in Kosambi map units (cM) of each LG are shown on the left, and the loci names are shown on the right. The LGs were grouped into homeologous pairs based on common homeologous loci connected by blue dashed lines. The polysomic and disomic markers are indicated in red and black colors, respectively. Duplicated loci are identified by the number 1 or 2 after the suffix A or B. The distorted loci are identified by asterisks after the locus name. The number of asterisks indicates the intensity of the distortion of segregation (^∗^*P* < 0.05, ^∗∗^*P* < 0.01, and ^∗∗∗^*P* < 0.001). The symbols after the asterisk specify the direction of the distortion of segregation < ′ >, < ″ >, and < ^′′′^ > indicate the distortion in favor of the cultivated genotype, heterozygous, and wild, respectively.

In our study, the genetic map was developed progressively due to the mixture in the dataset of codominant loci that segregated in a disomic or polysomic form, combined in some cases with the distortion of segregation. The map constructed without the distorted and the polysomic loci comprised 270 loci distributed on 16 LGs, while 34 loci remained unlinked. Distorted loci were then included in the map using LOD score values comprised between 4 and 7, *r* = 0.3. This allowed for the addition of two new LGs (LGB2 and LGB9) that were mainly formed by distorted loci (**Figure 2**). At this step, the map comprised 304 loci clustered in 18 LGs. In the third step, when adding the polysomic loci, two new LGs (LGA4 and LGB4) were formed and two initially distinct LGs (LGA3 and LGB3) clustered together. The clustered LGs were dissociated by removing three polysomic loci (Seq16F01, Ah3TC31H02, and Seq2H11). At this step, the resulted genetic map comprised 20 LGs, and 12 loci remained unlinked. The loci were then ordered within each LG. Finally, the estimated linkage map included 357 codominant loci distributed into 20 LGs and covering a total genetic distance of 1,728 cM, with an average interval of 5.1 cM between two adjacent markers.

We analyzed the distribution of the loci in the A and B/K-genomes. For the A-genome, 179 loci were mapped in 10 LGs with an average number of 18 markers per LG, ranging from 10 (LG A5) to 36 (LG A1). The length of the LGs ranged from 28.0 cM (LG A5) to 165.2 cM (LG A1), with an average of 104.3 cM. For the B/K-genome, 178 loci were mapped on 10 LGs with an average number of 18 markers per LGs ranging from 13 (LG B10) to 28 (LG B1). The length of the LGs ranged from 33.3 cM (LG B10) to 111.4 cM (LG B1), with an average of 68.5 cM (**Table 4**). The average interval between adjacent markers was 6.4 and 4.0 cM for the A- and B/K-genomes, respectively.

**Table 4:** Description of the genetic linkage map.

Linkage groups (LG)	Mapped loci	Total distance (cM)	Largest Gap (cM)	Mean interval (cM)	Homeologous recombination	
					Distance (cM)	Coverage (%)
A1	36	169.3	17.1	4.8		
B1	28	111.4	10.7	4.1		
A2	12	66.3	16.4	6.0		
B2	22	80.6	12.6	3.8		
A3	25	145.5	18.1	6.1	46.1	31.7
B3	20	67.4	8.9	3.5	21.4	31.7
A4	13	95.4	19.4	8.0	95.4	100.0
B4	14	65.9	15.3	5.1	65.9	100.0
A5	10	27.9	12.6	3.1		
B5	17	63.7	15.7	4.0		
A6	24	111.3	16.8	4.8	9.5	8.5
B6	20	78.8	12.3	4.1	14.9	18.8
A7	18	112.8	17.8	6.6	19.4	17.2
B7	15	72.9	15.8	5.2	17.9	24.6
A8	14	140.3	30.2	10.8		
B8	14	46.6	9.5	3.6		
A9	12	87.9	25.0	8.0		
B9	15	64.0	12.0	4.6		
A10	15	86.5	13.5	6.2		
B10	13	33.6	10.2	2.8		

Ten homeologous LGs were clearly identified based on the common homeologous loci (**Figure 2**). The number of bridge markers within each pair of homeologous LGs ranged from 3 to 16 (**Figure 2**). A good collinearity was observed among seven pairs of homeologous LGs (A2/B2; A3/B3; A4/B4; A5/B5; A7/B7; A8/B8; and A10/B10). Except for one major inversion on LG6, two local rearrangements on LG9 and three on LG1, a good synteny of the markers along the LGs was observed (**Figure 2**).

Except for three loci, all distorted loci (*P* < 0.05) were clustered along the LGs. Only 17 distorted loci were mapped in the A-genome, whereas 40 were mapped for the B genome. The distorted loci mapped in the A-genome were skewed toward the cultivated genotype (A4, A7, and A9 LGs), whereas those distorted in the B-genome were mainly skewed toward the wild genotype (B2 and B9 LGs) or the heterozygous genotype (B3, B4, B6, B7, and B10 LGs) (**Figure 2**). Overall, with few exceptions, we found that SD occurred in the regions that displayed homeologous recombination uncovering that homeologous pairing plays an important role in shaping SD in the tetraploid peanut genome.

### Distribution of Disomic and Polysomic Loci along the LGs

One of the most remarkable features of this map is the distribution of the disomic and polysomic loci along the LGs. Among the ten homeologous sets of LGs, six sets consisted of markers that segregated in a likely disomic pattern and three in a mixed pattern with the disomic and polysomic loci clustered on the same LG (**Figure 2**). Surprisingly, one set (LG4) consisted of markers that all segregated in a polysomic way. The percentage of polysomic loci along the LGs ranged from 13.3% (LG6) to 100% (LG4) with an average of 39.9%.

### Homeologous Recombination along the LGs and Homeologous Genome Substitution

**FIGURE 3:**
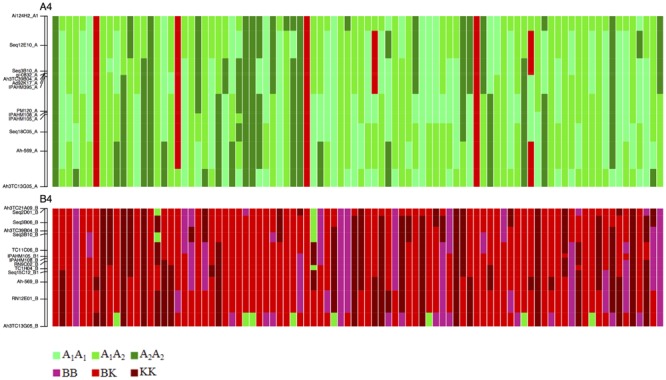
**Graphical genotype of the 90 F_2_ progeny at LG A4 and B4.** Loci name is shown on the left. Each column represents a F_2_ progeny. The green and red colors indicate the A and B/K genomes, respectively. The light-green, green, and dark-green colors indicate of the cultivated, heterozygous, and wild genotypes for A genome, respectively. The pink, red, and dark red colors indicate the cultivated, heterozygous and wild genotypes for the B/K genomes, respectively.

The co-inheritance of the polysomic loci allowed for the estimation of the portion of the genome that underwent homeologous recombination. These portions ranged from 14.9 cM for LG6 to 95.5 cM for LG4, with an average of 43.7 cM (**Table 3**). Our study clearly showed that for some progeny, the chromosomes were a mosaic of homologous and homeologous regions (**Figure 3**). Taking the physical distance covered by the LGs into account, a completed substitution of the A chromosome by its homeologous counterparts was observed in three F_2_ genotypes (**Figure 3**).

The location of the homeologous recombination breakpoints along the chromosomes was assessed for LG4, which displayed a full homeologous pairing. Of the 17 regions in which the homeologous exchanges occurred, 10 regions of the B-genome were replaced by the A corresponding region and the seven others exhibited the reciprocal situation (**Figure 3**). The number of the homeologous recombination breakpoints along the LGs 4A and 4B ranged from 1 to 3.

## Discussion

The segregation patterns of SSR loci in tetraploid peanut (2*n* = 4*x* = 40) were determined by studying their inheritance and by mapping genetic exchanges between homeologous genomes. Our results strongly support a mixed disomic and polysomic modes of genetic inheritance of SSR loci in the cross between a cultivated peanut variety and a synthetic allotetraploid. Mixed inheritance is rarely observed in tetraploid peanut and has not yet been reported when the AAKK and AABB genomes were involved.

### Evidence of Disomic and Polysomic SSR Inheritance

In our study, we showed that a large number of SSR markers were inherited likely in a disomic way, but some others showed genetic inheritance as a polysomic mode leading to a complex meiotic behavior.

Inheritance patterns of molecular markers have been considered a powerful method for determining meiotic behavior in polyploidy species (Lerceteau-Köhler et al., 2003) and have generated interesting conclusions about the genome behavior of several species including bermugrass (Guo et al., 2015), mimulus (Modliszewski and Willis, 2014), chrysanthemum (Klie et al., 2014), kiwifruit (Wu et al., 2013), roses (Koning-Boucoiran et al., 2012), yam (Bousalem et al., 2006; Nemorin et al., 2012), citrus (Kamiri et al., 2011), swithgrass (Okada et al., 2010), Yellow Cress (Stift et al., 2008), tomato (Barone et al., 2002), birdsfoot trefoil (Fjellstrom et al., 2001), sugar cane (Hoarau et al., 2001), and alfalfa (Diwan et al., 2000). The molecular methods used to distinguish disomy and polysomy are usually based on the signal intensity of PCR products and comparison of the number of loci linked in coupling versus the repulsion phase. However, the interpretation of multiple dose markers is often difficult in polyploids and is impossible in some species with polysomic inheritance (Esselink et al., 2004; Landergott et al., 2006).

In our study, to partially overcome the problem of complex electrophoresis profiles, we undertook a direct interpretation of SSR bands, suitably assigned to the subgenome of both parents. Using this approach, we observed that some markers followed the disomic inheritance as usually reported in allotetraploid peanut (Burow et al., 2001; Foncéka et al., 2009; Shirasawa et al., 2013) but some others exhibited an F_2_ genotype unexpected under disomic inheritance, but that fitted with a polysomic segregation. The exclusive presence of homologous alleles among some F_2_ genotypes for SSRs that mark homeologous genomes indicates that the homologous alleles ended up in the same gamete during the parental and/or the F_1_ meiosis. Our findings are consistent with the recent study published by Leal-Bertioli et al. (2015) that explained the missing data observed among genotypes by tetrasomic recombination.

In some cases, the segregation patterns of SSR loci were similar to that observed in case of double reduction. We observed a phenotypic class with only one allele in some F_2_ progeny, whereas up to 4 alleles segregate in the population. Double reduction refers to the fact that for a specific locus, the sister alleles come together in the same gamete during meiosis. It has been reported that the rate of double reduction is expected to increase towards the telomeres (Koning-Boucoiran et al., 2012; Bourke et al., 2015). In our study, the loci that showed this peculiar pattern of segregation are located at the extremity of LG3 (IPAHM103 and IPAHM282) and LG7 (RN13D04). However, since the genotype of the F_1_ plant that gave rise to the F_2_ progeny is unknown one cannot exclude that this pattern of segregation arose from the fusion of male and female gametes with the same haplotype (i.e., KK × KK).

### Mapping Genetic Exchanges between Homeologous Genomes

The construction of the linkage map allowed us to locate the regions where the genetic exchanges occurred between the homeologous genomes. The LGs 3, 4, 6, and 7, which underwent homeologous recombination in our study, are consistent with the findings of Leal-Bertioli et al. (2015). However, the percentage of markers involved in homeologous recombination was higher in our study compared to that mentioned in Leal-Bertioli et al. (2015) (11% vs. 3%). Moreover, in LG4, we report a complete substitution of the A-chromosome by its B/K homeologous genome in three progeny. In the study of Leal-Bertioli et al. (2015), the LG4 of the induced tetraploid parent was involved in an almost complete substitution of the B-chromosome by the A-chromosome. The similarity of the results between these two studies using different synthetic allotetraploids raised questions about the factors that drive homeologous recombination.

Pairing affinity between different sets of chromosomes was reported to be influenced by structural homology (Mason et al., 2010, 2014; Mandáková, Marhold and Lysak, 2014). Bertioli et al. (2016) reported a close similarity between the A and B wild species genomes based on sequence data comparison of *A. duranensis* and *A. ipaensis*. Moreover, in the present study and in many other genetic mapping studies in peanut (Burow et al., 2001; Foncéka et al., 2009; Shirasawa et al., 2013), a good collinearity was found between the homeologous genomes. These results are in favor of a homology-driven homeologous chromosome pairing.

However, we found some loci rearrangements between homeologous LGs that displayed a mixed inheritance. This was particularly the case for LG6, in which Bertioli et al. (2016) also reported a large inversion. Thus, there are probably others factors that drive the pairing between homeologous genomes in polyploid species. Those factors can be genetic (Cifuentes et al., 2010; Gaeta and Chris Pires, 2010), similar to that exerted by the *Ph-1* locus in wheat (Moore, 2014) in rye (Lukaszewski and Kopecký, 2010) and Brassica (Nicolas et al., 2009). More studies will be needed to decipher the molecular forces that drive homeologous pairing in peanut.

The results of this study suggest that, at least in an interspecific context, the meiotic pairing in tetraploid peanut fits with the intermediate inheritance where pairing of chromosome sets ranged from strict disomic inheritance as in diploids to full polysomic as in autopolyploids (Stebbins, 1947; Sybenga, 1996; Catalán et al., 2006; Xu et al., 2015). Many important plants are polyploids and some of them have been clearly identified as intermediate genetic pattern such as chrysanthemum (Klie et al., 2014), banana (Jeridi et al., 2012), and strawberry (Lerceteau-Köhler et al., 2003). These findings also raised the question of chromosome pairing during meiosis in crosses involving only cultivated peanut varieties. Until now, inter-genomic exchanges have never been reported using genetic mapping approaches in cultivated peanut. Recently, thanks to tetraploid versus diploid sequence comparisons, genomic exchanges between A and B sub-genomes have been reported (Bertioli et al., 2016). However, one still has to puzzle out whether these exchanges resulted from ancient recombination events that occured at the infancy of cultivated peanut as a consequence of the genomic shock due to the first hybridization between the A and B subgenomes or were due to something more recent that is still occurring in peanut varieties.

### Homeologous Recombination Occurred during the Meiosis of the Parents

The reconstruction of the genotype of the homeologous recombinants suggests that, homeologous pairings have arisen in the synthetic allotetraploid parent. Indeed, the genotype of some homeologous recombinant F_2_ progeny was either BKKK or BBKK. If homeologous recombination occurred only during the meiosis of the F_1_ plants and not in the meiosis of the parents, the genotype of the homeologous recombinant in the F_2_ progeny would only be A_1_A_1_A_2_A_2_ or BBKK (**Table 1**). The BKKK genotypic composition was regarded as a consequence of homeologous recombination during the meiosis of the parents particularly in the synthetic allotetraploid. In some species, homeologous pairing during meiosis is suspected to occur in the first generations following polyploid formation (Ramsey and Schemske, 2002; Soltis et al., 2010; Szadkowski et al., 2010).

### Implications for Peanut Breeding

The pattern of recombination between chromosomes of related species is a key point to transfer genes between the species. The knowledge about the inheritance mode is essential information not only because it sheds light on homeologous chromosome pairing behavior but can also influence the breeding strategies that are used for cultivar development. In this study, homeologous recombination events have been located in some genomic regions that are common with the ones reported in the study of Leal-Bertioli et al. (2015). One can suppose that these regions are particularly sensitive to homeologous recombination especially when synthetic allotetraploid are used. The SSRs that are located in these specific regions, particularly those that marked the homeologous genome in both parents, have proven to be very efficient in revealing the homeologous recombination events. Thus, they can be used to detect and trace back those events in the synthetic allotetraploid parental lines before crossing, as well as in their related F_1_ hybrids.

Mixed inheritance may have unwanted impacts on aberrant meiotic behavior, karyotype destabilization, and fertility reduction. However, it could also speed up the accumulation of rare but favorable alleles through homeologous recombination and marker-assisted introgression. The interspecific breeding population developed in this study would be an ideal genetic material to study the genetic effects of cumulative homologous and homeologous alleles and to transfer valuable alleles from wild species into cultigens. Despite conflicting results around the *A. batizocoi* taxon and its potential usefulness (Leal-Bertioli et al., 2014), its utilization for peanut breeding has been successful reported (Burow et al., 2014; Sukruth et al., 2015). Although erratic fertility was observed in some lines, the advanced backcross population developed from the same cross has a high phenotypic variation for many important agronomic traits, such as plant architecture, yield related traits, drought tolerance and resistance to leaf spot (Nguepjop et al., in preparation). We believe that the genetic variation among AB-QTL lines is increased when homeologous chromosomes pair. The mosaic compositions of the genome and the homeologous chromosome substitutions may speed up novel genetic combinations, opening new horizons for peanut breeding.

## Conclusion

The inheritance patterns of SSR markers, statistical analysis and genetic mapping provide evidence of a mixed disomic and polysomic mode of genetic inheritance in allotetraploid peanut based on an experimental interspecific cross. The mixed inheritance appears associated with segregation distortion and homeologous chromosome substitutions. These findings contribute to a better understanding of the meiotic behavior of allotetraploid peanut and will provide useful information to breeders that use synthetic tetraploid to move genes in the genetic background of the cultivated peanut species.

## Author Contributions

JN designed and coordinated the study, performed the experiments, carried out data analyses and map construction, and wrote the manuscript. H-AT was involved in population development. NM and SS have produced the synthetic amphidiploid used in the study. JB, BC, and DS were involved in the design of the study and helped in data analysis. J-FR designed the study involved in map construction and contributed to editing of the manuscript. DF conceived, designed, and coordinated the study, helped in data analysis and editing of the manuscript. All authors read and approved the manuscript.

## Conflict of Interest Statement

The authors declare that the research was conducted in the absence of any commercial or financial relationships that could be construed as a potential conflict of interest.

## References

[B1] Ainouche M. L. Wendel J. F. (2014). “Polyploid speciation and genome evolution: lessons from recent allopolyploids,” in *Evolutionary Biology: Genome Evolution, Speciation, Coevolution and Origin of Life* ed. Pontarotti P. (Berlin: Springer International Publishing) 87–113.

[B2] Barone A. Li J. Sebastiano A. Cardi T. Frusciante L. (2002). Evidence for tetrasomic inheritance in a tetraploid Solanum commersonii (+) *S. tuberosum* somatic hybrid through the use of molecular markers. *Theor. Appl. Genet.* 104 539–546. 10.1007/s00122-001-0792-1 12582656

[B3] Bertioli D. J. Cannon S. B. Froenicke L. Huang G. Farmer A. D. Cannon E. K. S. (2016). The genome sequences of *Arachis duranensis* and *Arachis ipaensis*, the diploid ancestors of cultivated peanut. *Nat. Genet.* 48 438–446. 10.1038/ng.3517 26901068

[B4] Bourke P. M. Voorrips R. E. Visser R. G. F. Maliepaard C. (2015). The double reduction landscape in tetraploid potato as revealed by a high-density linkage map. *Genetics* 201 853–863. 10.1534/genetics.115.181008 26377683 PMC4649655

[B5] Bousalem M. Arnau G. Hochu I. Arnolin R. Viader V. Santoni S. (2006). Microsatellite segregation analysis and cytogenetic evidence for tetrasomic inheritance in the American yam *Dioscorea trifida* and a new basic chromosome number in the Dioscoreae. *Theor. Appl. Genet.* 113 439–451. 10.1007/s00122-006-0309-z 16775695

[B6] Burow M. D. Simpson C. E. Starr J. L. Paterson A. H. (2001). Transmission genetics of chromatin from a synthetic amphidiploid to cultivated peanut (*Arachis hypogaea* L.). broadening the gene pool of a monophyletic polyploid species. *Genetics* 159 823–837. 11606556 10.1093/genetics/159.2.823 PMC1461827

[B7] Burow M. D. Starr J. L. Park C.-H. Simpson C. E. Paterson A. H. (2014). Introgression of homeologous quantitative trait loci (QTLs) for resistance to the root-knot nematode [*Meloidogyne arenaria* (Neal) Chitwood] in an advanced backcross-QTL population of peanut (*Arachis hypogaea* L.). *Mol. Breed.* 34 393–406. 10.1007/s11032-014-0042-2

[B8] Catalán P. Segarra-Moragues J. G. Palop-Esteban M. Moreno C. González-Candelas F. (2006). A bayesian approach for discriminating among alternative inheritance hypotheses in plant polyploids: the allotetraploid origin of genus borderea (Dioscoreaceae). *Genetics* 172 1939–1953. 10.1534/genetics.105.042788 16322527 PMC1456289

[B9] Cifuentes M. Grandont L. Moore G. Chèvre A. M. Jenczewski E. (2010). Genetic regulation of meiosis in polyploid species: new insights into an old question. *New Phytol.* 186 29–36. 10.1111/j.1469-8137.2009.03084.x 19912546

[B10] Diwan N. Bouton J. H. Kochert G. Cregan P. B. (2000). Mapping of simple sequence repeat (SSR) DNA markers in diploid and tetraploid alfalfa. *Theor. Appl. Genet.* 101 165–172. 10.1007/s001220051465

[B11] Esselink G. D. Nybom H. Vosman B. (2004). Assignment of allelic configuration in polyploids using the MAC-PR (microsatellite DNA allele counting—peak ratios) method. *Theor. Appl. Genet.* 109 402–408. 10.1007/s00122-004-1645-5 15085263

[B12] Favero A. P. Simpson C. E. Valls J. F. M. Vello N. A. (2006). Study of the evolution of cultivated peanut through crossability studies among *Arachis ipaensis*, A. duranensis, and A. hypogaea. *Crop Sci.* 46 1546–1552. 10.2135/cropsci2005.09-0331

[B13] Ferreira T. Rasband W. (2012). *ImageJ User Guide: IJ 1.42r*. Available at: https://imagej.nih.gov/ij/docs/guide/.

[B14] Fjellstrom R. G. Beuselinck P. R. Steiner J. J. (2001). RFLP marker analysis supports tetrasonic inheritance in *Lotus corniculatus* L. *Theor. Appl. Genet.* 102 718–725. 10.1007/s001220051702

[B15] Foncéka D. Hodo-Abalo T. Rivallan R. Faye I. Sall M. N. Ndoye O. (2009). Genetic mapping of wild introgressions into cultivated peanut: a way toward enlarging the genetic basis of a recent allotetraploid. *BMC Plant Biol.* 9:103 10.1186/1471-2229-9-103 PMC3091533 19650911

[B16] Fonceka D. Tossim H.-A. Rivallan R. Vignes H. Lacut E. de Bellis F. (2012). Construction of chromosome segment substitution lines in Peanut (*Arachis hypogaea* L.) Using a wild synthetic and QTL mapping for plant morphology. *PLoS ONE* 7:e48642 10.1371/journal.pone.0048642 PMC3501512 23185268

[B17] Gaeta R. T. Chris Pires J. (2010). Homoeologous recombination in allopolyploids: the polyploid ratchet. *New Phytol.* 186 18–28. 10.1111/j.1469-8137.2009.03089.x 20002315

[B18] Gaeta R. T. Pires J. C. Iniguez-Luy F. Leon E. Osborn T. C. (2007). Genomic Changes in Resynthesized *Brassica napus* and their effect on gene expression and phenotype. *Plant Cell* 19 3403–3417. 10.1105/tpc.107.054346 18024568 PMC2174891

[B19] Guo Y. Wu Y. Anderson J. A. Moss J. Q. Zhu L. (2015). Disomic inheritance and segregation distortion of SSR markers in two populations of *Cynodon dactylon* (L.) Pers. var. dactylon. *PLoS One* 10:e0136332 10.1371/journal.pone.0136332 PMC4546580 26295707

[B20] Haynes K. G. Douches D. S. (1993). Estimation of the coefficient of double reduction in the cultivated tetraploid potato. *Theor. Appl. Genet.* 85 857–862. 10.1007/BF00225029 24196060

[B21] Hoarau J.-Y. Offmann B. D’Hont A. Risterucci A.-M. Roques D. Glaszmann J.-C. (2001). Genetic dissection of a modern sugarcane cultivar (*Saccharum* spp.). I. Genome mapping with AFLP markers. *Theor. Appl. Genet.* 103 84–97. 10.1007/s001220000390

[B22] Hong Y. Chen X. Liang X. Liu H. Zhou G. Li S. (2010). A SSR-based composite genetic linkage map for the cultivated peanut (*Arachis hypogaea* L.) genome. *BMC Plant Biol.* 10:17 10.1186/1471-2229-10-17 PMC2835713 20105299

[B23] Hong Y. Liang X. Chen X. Liu H. Zhou G. Li S. (2008). Construction of genetic linkage map based on SSR markers in Peanut (*Arachis hypogaea* L.). *Agric. Sci. China* 7 915–921. 10.1016/S1671-2927(08)60130-3

[B24] Husted L. (1936). Cytological studies of the peanut *Arachis*. II. Chromosome number, morphology, and behavior and their application to the origin of cultivated forms. *Cytologia* 7 396–423. 10.1508/cytologia.7.396

[B25] Jeridi M. Perrier X. Rodier-Goud M. Ferchichi A. D’Hont A. Bakry F. (2012). Cytogenetic evidence of mixed disomic and polysomic inheritance in an allotetraploid (AABB) musa genotype. *Ann. Bot.* 110 1593–1606. 10.1093/aob/mcs220 23087127 PMC3503499

[B26] Kamiri M. Stift M. Srairi I. Costantino G. Moussadik A. E. Hmyene A. (2011). Evidence for non-disomic inheritance in a citrus interspecific tetraploid somatic hybrid between C. reticulata and C. limon using SSR markers and cytogenetic analysis. *Plant Cell Rep.* 30 1415–1425. 10.1007/s00299-011-1050-x 21409551

[B27] Klie M. Schie S. Linde M. Debener T. (2014). The type of ploidy of chrysanthemum is not black or white: a comparison of a molecular approach to published cytological methods. *Front. Plant Sci.* 5:479 10.3389/fpls.2014.00479 PMC4172100 25295046

[B28] Koning-Boucoiran C. F. S. Gitonga V. W. Yan Z. Dolstra O. van der Linden C. G. van der Schoot J. (2012). The mode of inheritance in tetraploid cut roses. *Theor. Appl. Genet.* 125 591–607. 10.1007/s00122-012-1855-1 22526522 PMC3397129

[B29] Kosambi D. D. (1943). The estimation of map distances from recombination values. *Ann. Eugen.* 12 172–175. 10.1111/j.1469-1809.1943.tb02321.x

[B30] Landergott U. Naciri Y. Schneller J. J. Holderegger R. (2006). Allelic configuration and polysomic inheritance of highly variable microsatellites in tetraploid gynodioecious *Thymus praecox* agg. *Theor. Appl. Genet.* 113 453–465. 10.1007/s00122-006-0310-6 16786342

[B31] Leal-Bertioli S. Shirasawa K. Abernathy B. Moretzsohn M. Chavarro C. Clevenger J. (2015). Tetrasomic recombination is surprisingly frequent in allotetraploid *Arachis*. *Genetics* 199 1093–1105. 10.1534/genetics.115.174607 25701284 PMC4391553

[B32] Leal-Bertioli S. C. M. Santos S. P. Dantas K. M. Inglis P. W. Nielen S. Araujo A. C. G. (2014). Arachis batizocoi: a study of its relationship to cultivated peanut (*A. hypogaea)* and its potential for introgression of wild genes into the peanut crop using induced allotetraploids. *Ann. Bot.* 115 237–249. 10.1093/aob/mcu237 25538110 PMC4551086

[B33] Leitch A. R. Leitch I. J. (2008). Genomic plasticity and the diversity of polyploid plants. *Science* 320 481–483. 10.1126/science.1153585 18436776

[B34] Lerceteau-Köhler E. Guérin G. Laigret F. Denoyes-Rothan B. (2003). Characterization of mixed disomic and polysomic inheritance in the octoploid strawberry (*Fragaria* × ananassa) using AFLP mapping. *Theor. Appl. Genet.* 107 619–628. 10.1007/s00122-003-1300-6 12768242

[B35] Lorieux M. (2012). MapDisto: fast and efficient computation of genetic linkage maps. *Mol. Breed.* 30 1231–1235. 10.1007/s11032-012-9706-y

[B36] Lukaszewski A. J. Kopecký D. (2010). The Ph1 locus from wheat controls meiotic chromosome pairing in autotetraploid rye (*Secale cereale* L.). *Cytogenet. Genome Res.* 129 117–123. 10.1159/000314279 20551609

[B37] Lyrene P. M. (2016). Phenotype and fertility of intersectional hybrids between tetraploid highbush blueberry and colchicine-treated *Vaccinium stamineum*. *HortScience* 51 15–22.

[B38] Mallikarjuna N. Senthilvel S. Hoisington D. (2011). Development of new sources of tetraploid *Arachis* to broaden the genetic base of cultivated groundnut (*Arachis hypogaea* L.). *Genet. Resour. Crop Evol.* 58 889–907. 10.1007/s10722-010-9627-8

[B39] Mandáková T. Marhold K. Lysak M. A. (2014). The widespread crucifer species *Cardamine flexuosa* is an allotetraploid with a conserved subgenomic structure. *New Phytol.* 201 982–992. 10.1111/nph.12567 24400905

[B40] Mason A. S. Batley J. Bayer P. E. Hayward A. Cowling W. A. Nelson M. N. (2014). High-resolution molecular karyotyping uncovers pairing between ancestrally related *Brassica* chromosomes. *New Phytol.* 202 964–974. 10.1111/nph.12706 24471809

[B41] Mason A. S. Huteau V. Eber F. Coriton O. Yan G. Nelson M. N. (2010). Genome structure affects the rate of autosyndesis and allosyndesis in AABC, BBAC and CCAB *Brassica* interspecific hybrids. *Chromosome Res.* 18 655–666. 10.1007/s10577-010-9140-0 20571876

[B42] Mather K. (1936). Segregation and linkage in autotetraploids. *J. Genet.* 32 287–314. 10.1007/BF02982683

[B43] Modliszewski J. L. Willis J. H. (2014). Near-absent levels of segregational variation suggest limited opportunities for the introduction of genetic variation via homeologous chromosome pairing in synthetic neoallotetraploid *Mimulus*. *G3* 20 509–522. 10.1534/g3.113.008441 PMC3962489 24470218

[B44] Moore G. (2014). The control of recombination in wheat by Ph1 and its use in breeding. *Methods Mol. Biol.* 1145 143–153. 10.1007/978-1-4939-0446-4_12 24816666

[B45] Muller H. J. (1914). A new mode of segregation in Gregory’s tetraploid primulas. *Am. Nat.* 48 508–512. 10.1086/279426

[B46] Nemorin A. Abraham K. David J. Arnau G. (2012). Inheritance pattern of tetraploid *Dioscorea alata* and evidence of double reduction using microsatellite marker segregation analysis. *Mol. Breed.* 30 1657–1667. 10.1007/s11032-012-9749-0

[B47] Nicolas S. D. Leflon M. Monod H. Eber F. Coriton O. Huteau V. (2009). Genetic regulation of meiotic cross-overs between related genomes in *Brassica napus* haploids and hybrids. *Plant Cell* 21 373–385. 10.1105/tpc.108.062273 19190241 PMC2660629

[B48] Okada M. Lanzatella C. Saha M. C. Bouton J. Wu R. Tobias C. M. (2010). Complete switchgrass genetic maps reveal subgenome collinearity, preferential pairing and multilocus interactions. *Genetics* 185 745–760. 10.1534/genetics.110.113910 20407132 PMC2907199

[B49] Preacher K. J. (2001). *Interactive Chi-Square Tests.* Available at: http://www.quantpsy.org/chisq/chisq.htm [accessed April 21 2016]

[B50] Qin H. Feng S. Chen C. Guo Y. Knapp S. Culbreath A. (2011). An integrated genetic linkage map of cultivated peanut (*Arachis hypogaea* L.) constructed from two RIL populations. *Theor. Appl. Genet.* 124 653–664. 10.1007/s00122-011-1737-y 22072100

[B51] Rami J.-F. Leal-Bertioli S. C. M. Foncéka D. Moretzsohn M. C. Bertioli D. J. (2014). “Groundnut,” in *Alien Gene Transfer in Crop Plants* Vol. 2 eds Pratap A. Kumar J. (New York, NY: Springer) 253–279.

[B52] Ramsey J. Schemske D. W. (2002). Neopolyploidy in flowering plants. *Ann. Review Ecol. Syst.* 33 589–639. 10.1146/annurev.ecolsys.33.010802.150437

[B53] Robledo G. Seijo G. (2010). Species relationships among the wild B genome of *Arachis* species (section *Arachis*) based on FISH mapping of rDNA loci and heterochromatin detection: a new proposal for genome arrangement. *Theor. Appl. Genet.* 121 1033–1046. 10.1007/s00122-010-1369-7 20552326

[B54] Salmon A. Flagel L. Ying B. Udall J. A. Wendel J. F. (2010). Homoeologous nonreciprocal recombination in polyploid cotton. *New Phytol.* 186 123–134. 10.1111/j.1469-8137.2009.03093.x 19925554

[B55] Seijo G. Lavia G. I. Fernandez A. Krapovickas A. Ducasse D. A. Bertioli D. J. (2007). Genomic relationships between the cultivated peanut (*Arachis hypogaea*, Leguminosae) and its close relatives revealed by double GISH. *Am. J. Bot.* 94 1963–1971. 10.3732/ajb.94.12.1963 21636391

[B56] Seijo J. G. Lavia G. I. Fernandez A. Krapovickas A. Ducasse D. Moscone E. A. (2004). Physical mapping of the 5S and 18S-25S rRNA genes by FISH as evidence that *Arachis duranensis* and A. ipaensis are the wild diploid progenitors of *A. hypogaea* (Leguminosae). *Am. J. Bot.* 91 1294–1303. 10.3732/ajb.91.9.1294 21652361

[B57] Shirasawa K. Bertioli D. J. Varshney R. K. Moretzsohn M. C. Leal-Bertioli S. C. M. Thudi M. (2013). Integrated consensus map of cultivated peanut and wild relatives reveals structures of the A and B genomes of *Arachis* and divergence of the legume genomes. *DNA Res.* 20 173–184. 10.1093/dnares/dss042 23315685 PMC3628447

[B58] Simpson C. E. (1991). Pathways for introgression of pest resistance into *Arachis hypogaea* L. 1. *Peanut Sci.* 18 22–26. 10.3146/i0095-3679-18-1-8

[B59] Simpson C. E. (2001). Use of wild *Arachis* Species/introgression of genes into *A. hypogaea* L. *Peanut Sci.* 28 114–116. 10.3146/i0095-3679-28-2-12

[B60] Simpson C. E. Krapovickas A. Valls J. F. M. (2001). History of *Arachis* including evidence of *A. hypogaea* L. Progenitors. *Peanut Sci.* 28 78–80. 10.3146/i0095-3679-28-2-7

[B61] Smartt J. Gregory W. C. Gregory M. P. (1978). The genomes of *Arachis hypogaea*. 1. cytogenetic studies of putative genome donors. *Euphytica* 27 665–675. 10.1007/BF00023701

[B62] Soltis D. E. Buggs R. J. A. Doyle J. J. Soltis P. S. (2010). What we still don’t know about polyploidy. *Taxon* 59 1387–1403.

[B63] Soltis D. E. Visger C. J. Soltis P. S. (2014). The polyploidy revolution then and now: stebbins revisited. *Am. J. Bot.* 101 1057–1078. 10.3732/ajb.1400178 25049267

[B64] Stalker H. T. (1991). A new species in section *Arachis* of peanuts with a D genome. *Am. J. Bot.* 78 630–637. 10.2307/2445084

[B65] Stebbins G. L. (1947). Types of polyploids; their classification and significance. *Adv. Genet.* 1 403–429. 20259289 10.1016/s0065-2660(08)60490-3

[B66] Stift M. Berenos C. Kuperus P. van Tienderen P. H. (2008). Segregation models for disomic, tetrasomic and intermediate inheritance in tetraploids: a general procedure applied to rorippa (Yellow Cress) microsatellite data. *Genetics* 179 2113–2123. 10.1534/genetics.107.085027 18689891 PMC2516083

[B67] Sukruth M. Paratwagh S. A. Sujay V. Kumari V. Gowda M. V. C. Nadaf H. L. (2015). Validation of markers linked to late leaf spot and rust resistance, and selection of superior genotypes among diverse recombinant inbred lines and backcross lines in peanut (*Arachis hypogaea* L.). *Euphytica* 204 343–351. 10.1007/s10681-014-1339-2

[B68] Sybenga D. J. (1975). “The analysis of chromosome pairing,” in *Meiotic Configurations Monographs on Theoretical and Applied Genetics* ed. Frankel R. (Berlin: Springer) 134–199.

[B69] Sybenga J. (1996). Chromosome pairing affinity and quadrivalent formation in polyploids: do segmental allopolyploids exist? *Genome* 39 1176–1184. 10.1139/g96-148 18469964

[B70] Szadkowski E. Eber F. Huteau V. Lodé M. Huneau C. Belcram H. (2010). The first meiosis of resynthesized *Brassica napus*, a genome blender. *New Phytol.* 186 102–112. 10.1111/j.1469-8137.2010.03182.x 20149113

[B71] van Berloo R. (2008). GGT 2.0: versatile software for visualization and analysis of genetic data. *J. Hered* 99 232–236. 10.1093/jhered/esm109 18222930

[B72] Varshney R. Bertioli D. Moretzsohn M. Vadez V. Krishnamurthy L. Aruna R. (2008). The first SSR-based genetic linkage map for cultivated groundnut (*Arachis hypogaea* L.). *Theor. Appl. Genet.* 118 729–739. 10.1007/s00122-008-0933-x 19048225

[B73] Wu J.-H. Datson P. M. Manako K. I. Murray B. G. (2013). Meiotic chromosome pairing behaviour of natural tetraploids and induced autotetraploids of *Actinidia chinensis*. *Theor. Appl. Genet.* 127 549–557. 10.1007/s00122-013-2238-y 24306317

[B74] Xu F. Tong C. Lyu Y. Bo W. Pang X. Wu R. (2015). Allotetraploid and autotetraploid models of linkage analysis. *Brief. Bioinform.* 16 32–38. 10.1093/bib/bbt075 24177380

[B75] Zhou X. Xia Y. Ren X. Chen Y. Huang L. Huang S. (2014). Construction of a SNP-based genetic linkage map in cultivated peanut based on large scale marker development using next-generation double-digest restriction-site-associated DNA sequencing (ddRADseq). *BMC Genomics* 15:351 10.1186/1471-2164-15-351 PMC4035077 24885639

